# Amaurosis Caused by a Carious Tooth

**Published:** 1861-01

**Authors:** 


### "AMAUROSIS CAUSED BY A CARIOUS TOOTH."

You might as well say, "Amaurosis caused by *having* teeth."

In the *Dental Cosmos* for November, 1860, on pages 199–200, may be found an extract with the above caption. And, as it is *foreign* original matter, of course, it must pass, whether it be sense or nonsense. The detail of the case clearly attributes the loss of vision, of thirteen months standing, not to a "carious tooth," but to a "splinter of wood," and goes on, very astutely, to *suppose* it came there by a mechanical force sufficient to make it traverse the "center of the tooth," (an upper molar.) Now, I thank both the Professor at Milna and the Professor at Philadelphia—each most heartily—*once*, and "F. P," as heartily, *three times*, (the exact number of times his initials occur in the detail) for the case, as it points a principle, and shows us that amaurosis can be produced by occult causes which, when removed, allow it to cease, in otherwise healthy subjects, which is very well for both novice and erudite dental surgeons to know. But as all cases are detailed for the purpose of instruction, let us not set down "remote, (the carious tooth) for approximate

(the splinter) causes, if we do not wish to befog and lead astray the needy simple ones we write to benefit. For, truth to say, but too few of us understand the significance of the accounts we are favored with from the medical journals, not only from the ambiguity of the articles themselves, but also, for the want of a knowledge of the parts affected, as to normal or abnormal physiological and pathological states, sufficiently accurate to enable us to criticise, intelligibly, the mere say so of any thing that comes to us from what we deemed a respectable source. It were better for us to stand still than to blunder in the dark, or better not to be led at all than to be inducted still further into the darkness. "Be *sure* you are right"—then advance. A.

